# A Formal Algorithm for Verifying the Validity of Clustering Results Based on Model Checking

**DOI:** 10.1371/journal.pone.0090109

**Published:** 2014-03-07

**Authors:** Shaobin Huang, Yuan Cheng, Dapeng Lang, Ronghua Chi, Guofeng Liu

**Affiliations:** College of Computer Science and Technology, Harbin Engineering University, Harbin, P. R. China; University of Michigan, United States of America

## Abstract

The limitations in general methods to evaluate clustering will remain difficult to overcome if verifying the clustering validity continues to be based on clustering results and evaluation index values. This study focuses on a clustering process to analyze crisp clustering validity. First, we define the properties that must be satisfied by valid clustering processes and model clustering processes based on program graphs and transition systems. We then recast the analysis of clustering validity as the problem of verifying whether the model of clustering processes satisfies the specified properties with model checking. That is, we try to build a bridge between clustering and model checking. Experiments on several datasets indicate the effectiveness and suitability of our algorithms. Compared with traditional evaluation indices, our formal method can not only indicate whether the clustering results are valid but, in the case the results are invalid, can also detect the objects that have led to the invalidity.

## Introduction

Clustering analysis attempts to discover distribution patterns of data objects [1]. Because only reasonable results are helpful to effectively further decision support, we should evaluate a clustering result before using it to judge whether it is reasonable. That is, we should confirm whether the clustering reflects the intrinsic character of the data. This evaluation can be called verifying the validity of clustering results. Visualization methods can intuitively reflect the validity of clustering results for two-dimensional data objects. However, it is difficult to present a visual description of high-dimensional data. In addition, although appropriate visualization tools can describe the data, it is also difficult to discern the cluster distribution in high-dimensional space. Therefore, we need a metric to evaluate the validity of the results of clustering analysis. Clustering analysis can be divided into crisp and fuzzy clustering according to the boundaries between clusters. Data objects in crisp clustering are divided into distinct clusters, where each object belongs to exactly one cluster; while the boundaries between objects in fuzzy clustering are un-sharp, so objects may belong to more than one clusters. Therefore, the evaluation methods of their validity are different. Some common evaluation indices in crisp clustering include external, internal, and relative indices [2]–[5]. The validity indices in fuzzy clustering take into account the degree to which an object belongs to one cluster, such as PC (Partition Coefficient) and PE (Partition Entropy) [6]. We will discuss the method of verifying the validity of crisp clustering results in this study.

External indices are based on a priori partition information of data. These indices evaluate the validity of clustering results by matching the cluster structures and the priori information; the F-Measure, Jaccard, Purity and Entropy are all external indices [7]–[9]. In most research on clustering algorithms, the authors choose these external indices to evaluate the validity because of their intuitive nature. A comparison of some common external indices is shown in Table 1. As seen in the comparison, although the definitions of these indices are different, their basic ideas are all to compare the fit between clusters and the priori partition. If we specify a threshold for the fit, we can conclude whether a clustering result is valid by comparing its corresponding index value with the threshold, as explained in the Experimental Section. Obviously, the premise of applying an external index requires a priori partition information of the dataset. Therefore, there is no value in using external indices without partition information. In many practical applications, partition information for the data is not available, and thus, external indices cannot be used to evaluate clustering quality.

**Table 1:** Comparison of External Indices.

Evaluation Index	Implication	Formula	Notes
Purity	Reflects the purity of objects in clusters	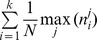	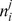 is the number of objects in the *i*th cluster belonging to the *j*th category
Entropy	Reflects the confounding, or impurity, of objects in clusters	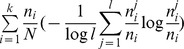	See Purity
Rand	Measures the similarity between clustering result C and the a priori partition P	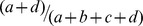	a, b, c and d indicate the number of object pairs in SS, SD, DS and DD, respectively,and *a+b+c+d = N(N−1)/2*
Jaccard	Measures the similarity between C and P	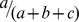	See Rand
FM	Measures the similarity between C and P	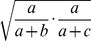	See Rand

*D* indicates the dataset containing *N* data objects, and its a priori partition with *l* categories is 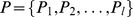. The result obtained from clustering is 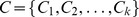, where *k* is the number of clusters. SS indicates that both objects belong to the same cluster of *C* and to the same group of partitions *P*; SD indicates points that belong to the same cluster of *C* and to different groups of *P*; DS indicates points that belong to different clusters of *C* and to the same group of *P*; and DD indicates points that belong to different clusters of *C* and to different groups of *P*.

An internal evaluation index evaluates clustering results using quantities and features inherent in the dataset, i.e., it assesses the fit between the clusters and the data using only the data themselves, rather than with a priori partition information [10], [11], such as the Cophenetic Correlation Coefficient and Hubert's 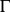 statistic, etc.. Table 2 compares several internal indices. It is obvious that a priori partition information is not necessary for internal indices. However, the above internal indices are only appropriate for hierarchical clustering or a single clustering scheme. In addition, they require relatively high computational complexity due to statistical testing.

**Table 2:** Comparison of Internal Indices.

Evaluation Index	Implication	Formula	Notes
CPCC	Measures the similarity between matrix Pc and P, where Pc(i,j) is the similarity of x_i_ and x_j_ when they are assigned to the same cluster, and P is an adjacency matrix	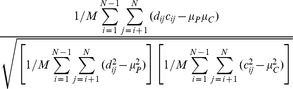 , where *N* is the number of objects, and 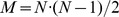 , 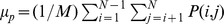 , 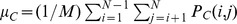 , 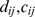 represent the elements in P and Pc, respectively.	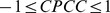 . This is appropriate for hierarchical clustering.
Hubert's 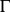	Measures the similarity between a clustering result C and the proximity matrix P	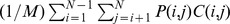 , where 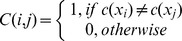	The larger the value, the higher the similarity between P and C.
Normalized 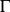	Measures the similarity between a clustering result C and the proximity matrix P	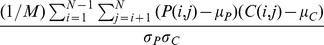	The larger the value, the higher the similarity between P and C.

Relative indices are different from the above validation methods and are used to choose the best clustering scheme from a set of defined schemes according to a pre-specified criterion [12]–[14], i.e., they compare a clustering scheme with other clustering schemes based on indices such as the Dunn index, the Davies-Bouldin index or the SD validity index. Table 3 compares some common relative indices; note that most are based on the separation and compactness of clusters. Relative indices are used to compare the relative performance of several clustering schemes rather than to verify the validity of a clustering result explicitly, as external indices do. Thus, it is difficult to infer whether a clustering scheme is reasonable or valid directly without comparison with other clustering schemes, such as sorting on the index values of different clustering schemes, which will also be explained in the Experimental Section. In addition, clustering algorithms assign objects to clusters based on distances between the objects, while relative indices are almost always defined based on distances. If the relative indices are still based on distances, and the definition of distance is itself imprecise, we can obtain only a relative evaluation result.

**Table 3:** Comparison of Relative Indices.

Evaluation Index	Implication	Formula	Notes
Dunn	Measures the compactness of clusters and separation between clusters	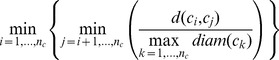	The larger the value, the better the clustering effect
DB	Measures the compactness of clusters and separation between clusters	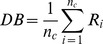 _,_ ( 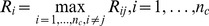 ; 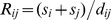 )	The smaller the value, the better the clustering effect
RMSSDT	Measures the differences between clusters	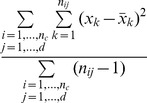	The smaller the value, the better the clustering effect
SD	Measures the compactness of clusters and separation between clusters	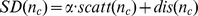 , 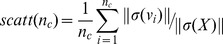 , 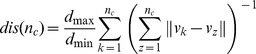	The larger the value, the better the clustering effect
S_Dbw	Measures the intra-cluster variance and inter-cluster density	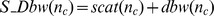 , 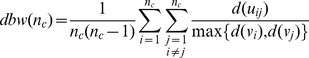	The smaller the value, the better the clustering effect

Although the evaluation methods mentioned above are generally used to assess the validity of clustering results, it is evident that these methods all have limitations. We feel that these limitations will remain difficult to resolve if the verification method continues to be based on clustering results and evaluation index values. While recent studies have proposed new evaluation methods, some of these methods are still based on clustering results [15], [16] or distances between objects [17], [18]; these methods may continue to encounter the above limitations. Other new methods focus on specific applications, such as clustering related to prediction [19], wireless sensor networks [20], and natural language processing [21].

Therefore, this study proposes a formal algorithm to verify the validity of clustering results and to avoid the above limitations. We focus on clustering processes, trying to verify the validity of the clustering results by analyzing whether a clustering process satisfies corresponding properties. First, clustering is a type of unsupervised machine learning method. Regardless of the type of clustering algorithm, most explore data concentration during iterative processes based on the distances between objects. Therefore, we can extract a model to describe the iterative processes that reflect the essence of clustering processes even with many different clustering algorithms. Furthermore, we use evaluation indices reflecting cluster separation and compactness to measure the effectiveness of every clustering iteration. As mentioned in Section 2.3, the processes yielding valid clustering results will satisfy such properties: evaluation indices gradually increase (or decrease) with each iteration in clustering processes. If a clustering process does not satisfy predefined properties, we can conclude that its result will fail to discover the distribution pattern of data objects, i.e., its result will not satisfy a validity test.

As an automatic formal verification method, model checking can describe system behaviors as a state transition system T and the properties 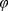 systems should obey by using LTL (Linear Temporal Logic) [22] or CTL (Computation Tree Logic) [23]. We can then recast the problem of verifying whether a system has specific properties as a mathematics problem to be solved by mechanical steps, i.e., verifying whether T satisfies 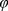. Based on the correspondence of clustering to model checking, we can recast the problem of judging the validity of a clustering result as verifying whether the model describing a clustering process satisfies specific properties. Therefore, we propose a general formal method of verifying the validity of clustering results based on model checking. The method can determine whether a clustering result is reasonable and valid. When the result is invalid, the method can also use counterexamples to determine which iterations cause the invalidity when applying a clustering algorithm to a dataset. The method can even identify the objects that affect the validity.

First, we define the abstraction method of modeling a clustering process, explain its operational semantics and extract criteria for judging the validity of clustering results as CTL formulas, i.e., properties satisfied by a valid clustering result. We then propose a formal method to verify whether a clustering process satisfies the specific properties based on model checking. Furthermore, if the process does not satisfy the validity test, we will use the previous verification results to find the objects that may generate the invalid result.

The remainder of this paper is organized as follows. We first discuss the abstract method of modeling clustering processes and representing the properties to be verified. We then propose the formal algorithm for verifying the validity of clustering results based on model checking and present the experimental results. Finally we conclude the study.

## Methods

### Model checking

**Figure 1:**
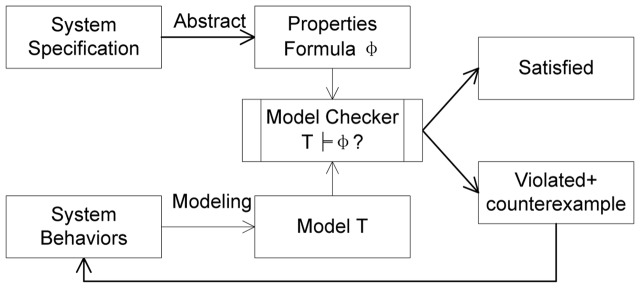
Workflow of model checking.

Hardware and software systems are all designed and implemented by humans, for whom it is impossible not to make mistakes. These mistakes imparted to systems always disturb humans, even after a complete testing. Therefore, even as people are enjoying the efficiency and convenience of information technology, they are also taking risks that the hardware or software systems will fail. How to ensure the correctness and dependability of the hardware and software systems has become a serious problem. To verify the correctness and dependability of a system, during the 1980s, Clarke and Quielle proposed model checking techniques based on temporal logic [24]. Model checking is an automatic, model-based method of verifying properties; its basic idea is shown in Figure 1. The transition system T indicates the system behaviors, and an LTL or CTL formula 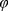 describes the system specification. Thus, the problem of judging whether a system has the expected specification can be recast as verifying whether the transition system T satisfies formula 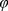; formally, T| = 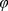
?. This problem is decidable when the transition system is a finite state transition system, i.e., the model checking algorithm will either terminate with the answer “true”, indicating that the model satisfies the specification, or give a counterexample execution that shows why the formula is not satisfied [25].

The model checking process has three phases: a modeling phase, a checking phase and an analysis phase.

#### (1) Modeling phase

We should describe the two necessary inputs of model checking, i.e., the system model and its properties, precisely and unambiguously. A finite state automaton is always used to model systems. The automaton consists of a set of states and a set of transitions. States are used to compare the current variable value with the prior value, while the transition set describes how the system moves from one state to another. Modal logic is always used to formalize system properties. Because temporal logic is an extension of propositional logic, it incorporates operators describing the temporal characteristics of system behaviors and can describe system properties such as correctness (whether a system completes prospective functions), accessibility (whether a system terminates at the deadlock state), security (unexpected behaviors will never happen), activity (expected behaviors will eventually happen) and fairness (whether an event will continue to recur in certain conditions).

#### (2) Checking phase

Checking involves first initializing every variable, setting and instruction, then verifying the specific properties with model checking procedures. The verification process explores all of the states in the system model to detect states that violate specific properties.

#### (3) Analysis phase

The two possible results of verification are that the model satisfies the properties or that it violates them. There are two reasons for not satisfying properties. One is model error, in which case we should refine the model and properties and then repeat the entire process; the other is design error, in which case the verifying results can be considered as a counterexample and used to revise the design of the system.

Because of its automation and the ability to localize the causes of errors, model checking has been used to verify the design of some hardware and software, such as to verify the IEEE Futurebus+ cache coherence protocol and to find a number of previously undetected errors in the design of the protocol [26]; to debug a hardware laboratory failure in the PowerPC 620 microprocessor during the booting of an operating system and to find a BIU deadlock causing this failure [27]; and to check the properties of the AT-&-T High-level Data Link Controller design and to find a bug that would have reduced throughput or caused lost transmissions [28], among other problems.

As mentioned in the Introduction, we will use model checking to verify the clustering results based on the correspondence of clustering to model checking. Therefore, we will follow the three phases of model checking and begin by formally describing the clustering processes and the properties satisfied by valid clustering results in the subsequent sections.

### Abstract modeling of clustering processes

Crisp clustering algorithms can be divided into categories such as partitioning methods, hierarchical methods, density-based methods, model-based methods, and SVM-based methods etc. As noted in the Introduction, clustering is an unsupervised machine learning method. Therefore, to ensure clustering quality, most of these algorithms need multiple iterations, although the clustering processes of these algorithm categories different from one another. The differences are the ways in which they group data. For example, K-Means adjusts the clusters of data objects in each iteration, DBSCAN grows clusters by gathering density-reachable objects directly from a core object in each iteration, a hierarchical clustering algorithm may merge or divide clusters in each iteration, while a neural network-based clustering method [29] adjusts the model vector to match objects in each iteration. We can then abstract and model such algorithms of different types based on the iterations of their clustering processes. In addition, employing different algorithms on the same dataset and the same algorithm on different datasets may result in different levels of clustering effectiveness. Therefore, we will formally describe the clustering process of any clustering algorithm applied to a dataset.

In model checking, transition systems are often used as models to describe the behavior of various systems, such as sequential hardware circuits, serial software programs, and parallel systems [30]. They are directed graphs whose nodes represent states and whose edges denote the state transitions. A state describes information of the system behaviors at a given moment. For example, the state of a program indicates the current values of its variables. The transitions between states are caused by actions and indicate the changes of variable values. For a data-dependent system, the state transitions may be caused by the action together with the characteristics of the system variables. For instance, the clusters will continue to adjust if the clustering is not convergent, and the clusters will not change once the clustering is convergent. To make the transitions deterministic without conditions in transition systems, we will first formalize them by means of a program graph over a set of variables, which is also a directed graph whose edges are labeled with conditions on these variables and actions [30]. Then, a transition system can be obtained by unfolding the program graph and can be used for the model in model checking.

Therefore, we build the program graph of a clustering process first to describe the changes of variable values with a clustering process, as described in Definition 1:

Definition 1. The program graph (PG) for a clustering process is a six-tuple (*Loc*, *Act*, *Effect*, →, *Loc*_0_, *g*_0_), where:

1. *Loc* is a set of locations in which every location 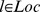 corresponds to a variable evaluation set 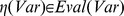, and 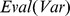 denotes the set of evaluations assigning values to every variable in *Var*.
2. *Act* is a set of actions in clustering.

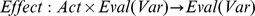
 is the effect function and indicates how the evaluation of variables is changed after performing an action.

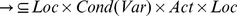
 is the set of conditional transition relations. In a conditional transition 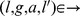, *g* is the guard of the conditional transition, e.g., the Boolean variable indicating whether it is true that the clustering is convergent. Then, the behavior of location 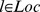 depends on the current variable value *η*. That is, when *η* satisfies condition *g*, the execution of action *α* will change the evaluation of variables according to *Effect*(*α*, *η*); subsequently, the system changes to location *l*′.

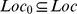
 is a set of initial locations.

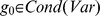
 is the initial condition.

Based on the essentials of the clustering algorithms mentioned above, we note that there are two core operations in clustering processes: comparing distances between objects (*compare*) and assigning objects into clusters (*allocate*). If these are the actions in a program graph, a clustering process can be regarded as multiple iterations consisting of these two actions and the evaluations of variables. Obviously, the program graph is not appropriate for clustering algorithms that generate clusters without iterations. For example, an SVM-based clustering method [31] performs clustering by using constrained nonlinear programming methods, which can solve problems by KKT conditions and do not need multiple iterations; consequently, we cannot obtain its program graph based on the above idea. The program graph is also not appropriate for the algorithms with probability due to the restriction of the domains of the variables and the effect of actions in the program graph. The probability will cause states to be infinite, which will affect the decidability of model checking. For example, although the EM (Expectation Maximization) method needs multiple iterations to ensure the clustering accuracy, we also cannot obtain its program graph because of its probabilistic nature.

**Figure 2:**
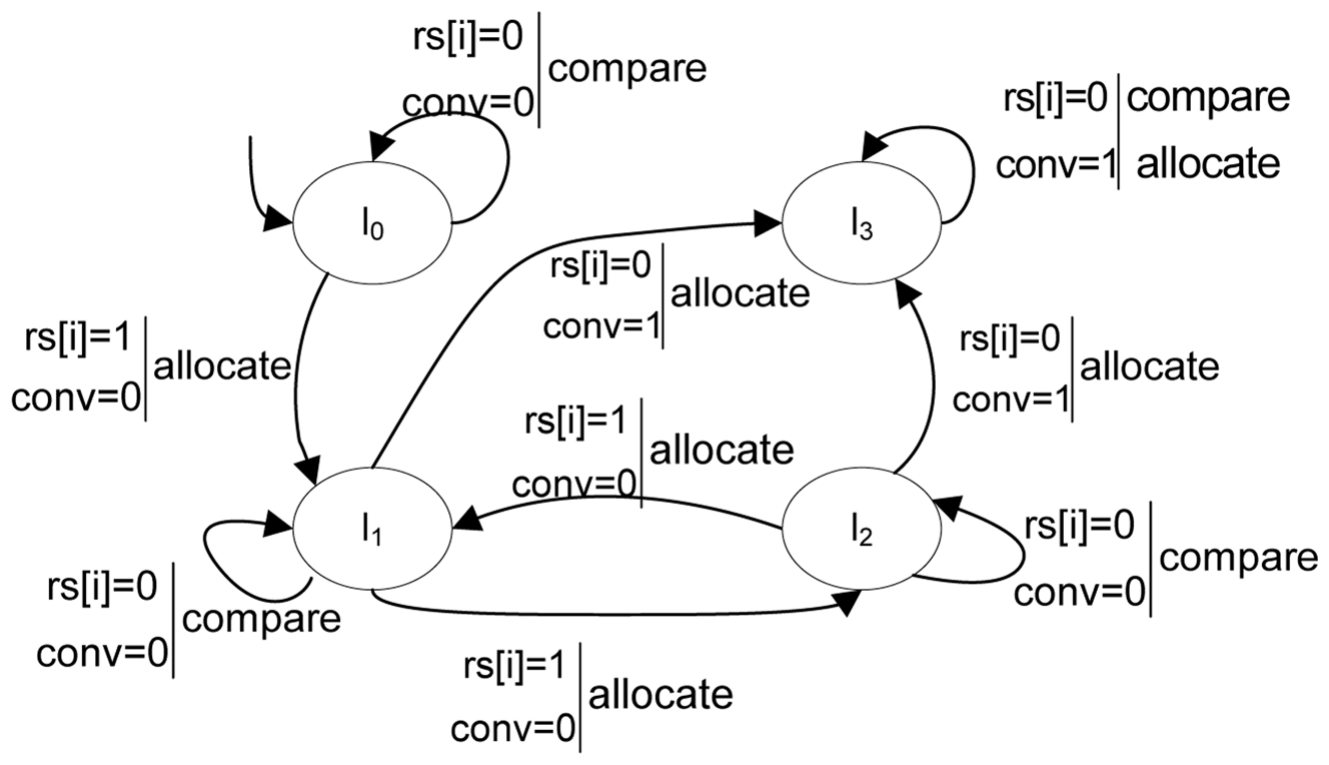
Program graph for the execution of K-Means.

Then, we will use the executive process of K-Means as an example to illustrate its corresponding program graph. Figure 2 describes its graphical representation.

1. Variable set 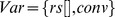, in which array *rs* registers whether the objects in each cluster have changed. The domain of 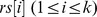 is 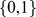, where *k* is the number of clusters; 0 indicates the objects in the *i*th cluster have not changed, while 1 indicates they have changed. *conv* indicates whether the clustering converges and, hence, terminates. In K-Means, for example, *conv* is the difference of the criterion function between any two iterations, and its domain is {0,1}, where 0 is not convergent, and 1 is convergent.
2. *Act* = { *compare*, *allocate* }.
3. *η* is the effect on variable evaluation induced by the action in *Act*, which is defined based on the semantics of clustering processes:
*Effect*(*compare*, *η*) = *η*, indicates that none of the variable values will change after the “*compare*” action.
*Effect*(*allocate*,*η*(*conv = 0*)) = *η*(*rs*[*i*] = 1), where 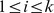. In K-Means, if the clustering is not convergent, the objects in a cluster will change after the “*allocate*” operation.
*Effect*(*allocate*,*η*(*conv = 1*)) = *η*(*rs*[*i*] = 0), where 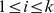. Here, once the clustering is convergent, the clusters will not change.
4. The initial condition of clustering is that none of the objects have been disposed of, and there is no object in any cluster, i.e., *g*_0_ = ( *rs*[*i*] = 0; *conv* = 0), where 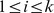.
5. The conditional transitions indicate the transitions of locations that meet the conditions. In K-Means, they include the following transitions, the action ‘*allocate*’ will cause the location meeting the initial condition to transfer a new location; if *conv* is 0, the ‘*compare*’ action will not cause the change of a location because the clusters do not change and the ‘*allocate*’ action will make the change of locations; and if *conv* is 1, the locations will not change after either the ‘*compare*’ or ‘*allocate*’ action.

**Figure 3:**
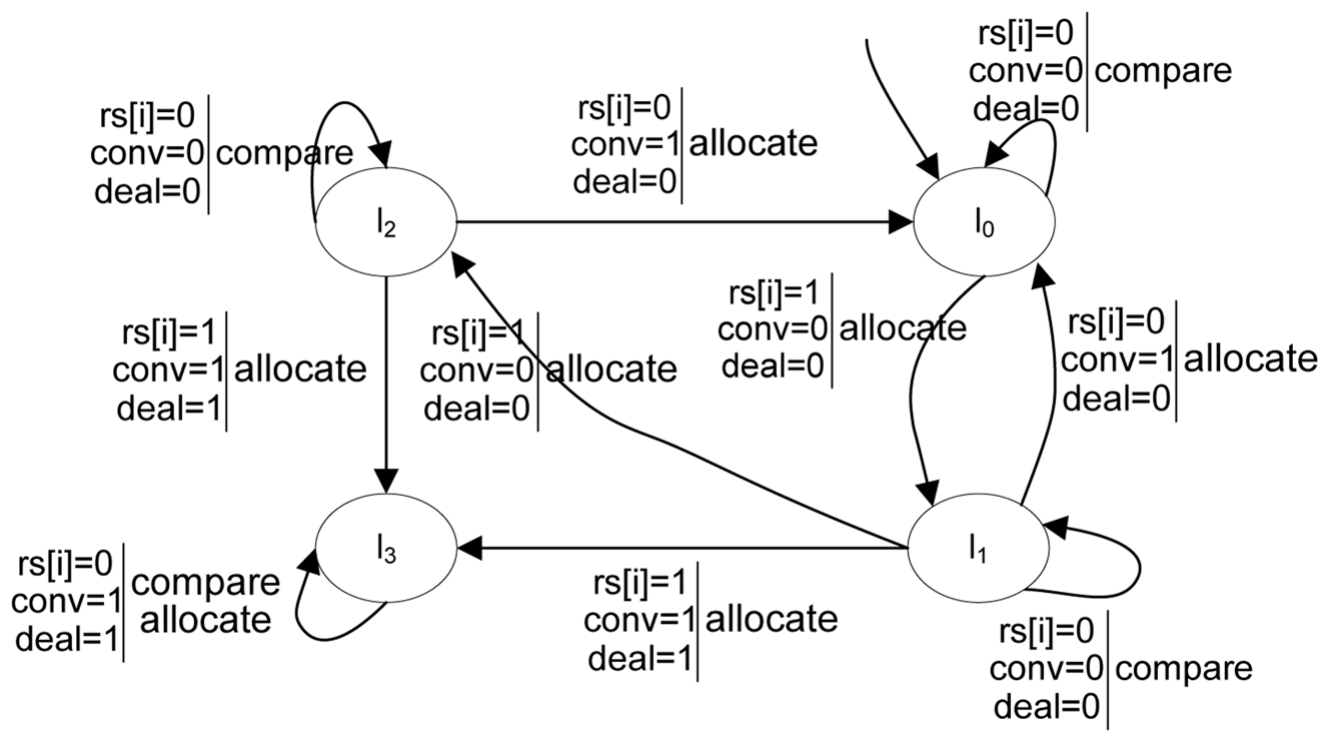
Program graph for the execution of DBSCAN.

Furthermore, different categories of clustering processes have different program graphs due to their different ways of grouping clusters, and the differences are mainly reflected in the variables and their evaluations of variables implicated by an action along with the conditional transition relations. As a comparison, the program graph of DBSCAN is shown in Figure 3. This program graph is different from K-Means, whose objects are all disposed of after allocating in each iteration. In Figure 3, a new core object is disposed of to find its density-reachable objects in each iteration. Therefore, the variables have been 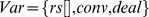, where *conv* indicates whether all of the density-reachable objects of one core object has been found, *deal* indicates whether the objects are all allocated, and their domains are {0,1} with the same meaning as the above. If *deal* is 0, ‘*allocate*’ will make the change of locations; if *conv* is 1 and *deal* is 0, it will start a new iteration to dispose of a new core object; once all objects have been allocated, i.e. *conv* is 1 and *deal* is 1, the locations will not change after either the ‘*compare*’ or ‘*allocate*’ action.

The program graph describes the changes of variable values that are impacted by action and the conditions over variables in a clustering process. Subsequently, we will interpret the semantics of a program graph using a transition system. The transition system represents the behaviors of a clustering as a state transition diagram, and can be obtained by unfolding the program graph then defined as the abstract model of a clustering process, i.e., as the verification system model of model checking.

Definition 2. The transition system of a program graph describing a clustering process is a six-tuple with a Kripke frame, TS(C) = (*S*, *Act*, *δ*, *I*, *AP*, *L*), where:

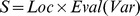
 is the finite set of system states. Every state consists of a location *l* of the program graph and the evaluation *η* of all variables. The initial state is the location satisfying initial condition *g*_0_.
2. *Act* is the finite set of actions that are the operations in clustering.

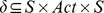
 is the set of state transition relations.

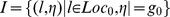
 is the set of initial states.
5. *AP* = *Loc*∪*Cond* (Var) is the set of atomic propositions. The atomic propositions formally express the characteristics of a clustering.

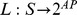
 is the label function, i.e., 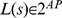, and it maps a set of atomic propositions to any state 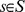. For a given logical formula φ, if the atomic propositions in 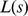 hold the formula φ, it can be said that state *s* satisfies φ, formally, 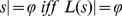.

Each state in a transition system corresponds to a set of evaluations of variables. The variables also include some Boolean variables as atomic propositions, such as *maxDunn*, which will constitute the property formulas to be satisfied by a valid clustering process.

**Figure 4:**
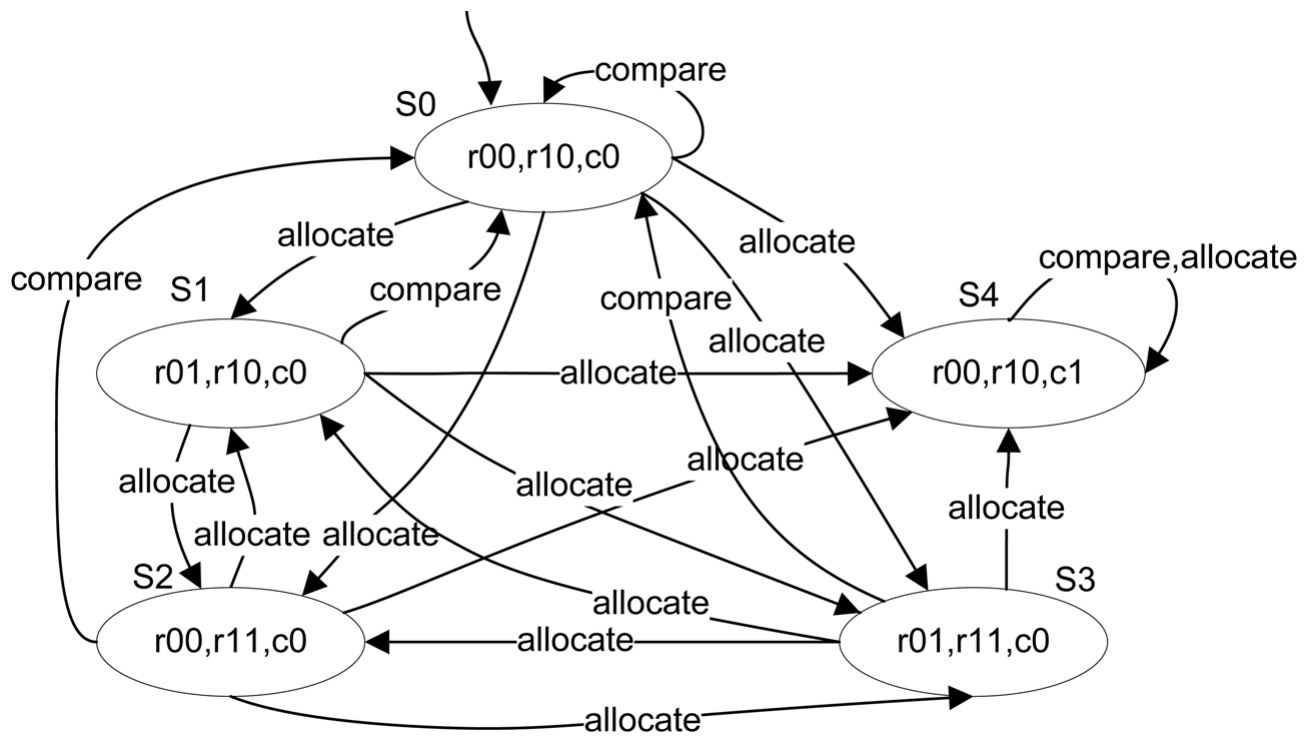
State transition diagram for the execution of K-Means on bankdata dataset.

We then take K-Means applied to the bankdata dataset as an example. Its transition system is presented in Figure 4. We ignore the atomic propositions and set the number of clusters as two, then a state consists of three variables and their evaluations, such as ‘*r_01_,r_10_,c_0_*’ in state *S_1_* indicates that the objects in cluster 0 have changed while those in cluster 1 have not changed, and the clustering is not convergent. The transition system depicts the changes of variable evaluation with the execution of an action, and the state transitions reflect the idea of clustering algorithms. The actual clustering process is one path in this transition system. In addition, the atomic propositions can be used to formulate the relevant clustering properties. Therefore, a transition system can be used as the verification system model of model checking.

If we want to verify the clustering validity with model checking methods, we should also formally describe the properties that valid clustering processes should satisfy. These property formulas consist of atomic propositions in Definition 2. In the next section, we will discuss the method of describing the properties with CTL formulas and their semantics in a transition system model as the basis of subsequent verification algorithms.

### Property formulas of validity

Model checking requires that the formal description of the system model and the properties be verified. The formal modeling methods of clustering processes are described above. This section will discuss the methods of representing property formulas satisfied by a valid clustering process.

As the expansion of classical logic, LTL and CTL both include temporal operators and both can be used to represent the properties to be verified. The former quantifies universally over paths in the model, while the latter includes the quantifiers A and E to respectively express the “all paths” and “existing one path” as well as the temporal operators in LTL. For example, CTL can express the semantic describing that there is a reachable state satisfying the property *p* or that whenever a state satisfying property *p* is reached the system can satisfy the property *q* continuously forever. A process of applying a clustering algorithm over specific parameters on a dataset is a path in a state transition diagram. Accordingly, we will describe the property specifications that a valid clustering process should satisfy based on CTL formulas along with a consideration of the balance of its expressive power and moderate decision procedure complexity [30].

The CTL formulas are presented in Appendix S1, whose detailed explanations can be found in [23]. In addition, there is redundancy between the connectives in CTL formulas in Appendix S1, and we can reduce the number of categories of verification algorithms by analyzing the equivalence relations with each other. As in [32], an adequate set of CTL is a subset of temporal connectives in CTL that is sufficient to express the equivalent formulas in CTL. An adequate set of temporal connectives in CTL contains at least one of {AX, EX} and one of {EG, AF, AU}, as well as EU. A common adequate set of CTL is {AF, EU, EX}. Then, the CTL formulas can be described as Appendix S2. The CTL model checking algorithms can be designed based on the connectives in an adequate set.

After the clustering processes, the clusters should be widely spaced, i.e., the separation between clusters should be maximized, while the members of each cluster should be as close to each other as possible, i.e., the compactness in clusters should be minimized. Therefore, a valid clustering result indicates that the result fits the inherent characteristics of the data better based on evaluation indices, i.e., for an external index, its value should be beyond the threshold; and for a relative index, its corresponding separation should be larger and compactness should be smaller as much as possible. A clustering process yielding a valid result can be seen as valid. In the other words, a valid clustering process can produce a valid clustering result. We will exploit these ideas to depict the properties satisfied by a clustering process that produces valid clustering results. Therefore, we develop the relationship between evaluation indices and valid clustering processes as described in Property 1 first.

Property 1. The values of evaluation indices will gradually increase (or decrease) with every iteration in a valid clustering process.

Consider an evaluation index denoting the comparison of separation between clusters and compactness in clusters. We suppose that the larger its value is, the better the clustering effect is. If the value decreases with an iteration of a clustering process, it is obvious that the algorithm to be verified fails to discover the distribution pattern of the dataset effectively. That is, the data do not tend to generate valid clusters with that clustering process. The decreasing index value indicates that the objects within a cluster do not have high similarity to one another and are not very dissimilar to objects in other clusters. Similarly, the indices in which a smaller value indicates a better clustering effect also satisfy this property.

We can now write Definition 3 to define the formula set described by CTL based on Property 1, whose formulas indicate the properties satisfied by valid clustering processes.

Definition 3. If indices 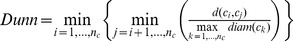 and 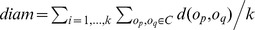 are used to define valid properties, the CTL formula set of properties that valid clustering processes should satisfy is as follows:

1. EF [ *converge* ∧ *handled*→*maxDunn* ∧ *minDiam* ],
2. E[(*DunnUp* U *maxDunn*)]∧ E[(*diamDown* U *minDiam*)],

where *converge* is the atomic formula to judge whether the clustering converges, the atomic formula *handled* judges whether all objects are disposed of by the algorithm, *maxDunn* is used to judge whether the *dunn* index reaches a maximum value, *DunnUp* is the atomic formula to judge whether the *dunn* index value increases, *diamDown* is used to judge whether the *diam* index value decreases, and the atomic formula *minDiam* judges whether the *diam* index reaches a minimum value.

We choose two indices, “*Dunn*” and “*diam*”, to define the property formulas in Definition 3. First, based on Property 1, these formulas include both the index increasing with the clustering process and the index decreasing with the clustering process to well reflect the relationship between evaluation indices and valid clustering processes. Secondly, we should choose indices based on lower computational complexity to facilitate the formal verification rather than on partition information. Next, because relative indices can be used to compare different clustering results, they can also be used to compare different iterations in a clustering process. Therefore, the common relative indices “*Dunn*” and “*diam*” are more appropriate and sufficient to describe the properties satisfied by a valid clustering process.

We will then analyze these formula semantics in the transition system model that will be used for verifying whether the model satisfies the properties described by CTL formulas with model checking. If the model satisfies the formulas, we can conclude that the corresponding clustering process is valid and, thus, that its clustering result is also valid. The transition system model consists of states and state transition relations that describe a clustering process. The semantics of the formulas in Definition 3 in the transition system model are as follows:

1. M, s| = EF [ *converge* ∧ *handled*→*maxDunn* ∧ *minDiam*] holds iff. there exists a trace started from state *s* in M and in some state in the future. When *converge* and *handled* are true, *maxDunn* and *minDiam* are also true, i.e., when the clustering algorithm converges and all objects are disposed of, the evaluation index *Dunn* reaches a maximum value, and the compactness in clusters *diam* reaches a minimum value.
2. M, s| = E[(*DunnUp* U *maxDunn*)]∧ E[(*diamDown* U *minDiam*)] holds iff. there exists a trace from state *s* in M satisfying that *DunnUp* stays true until *maxDunn* is true and *diamDown* stays true until *minDiam* is true, i.e., the value of index *Dunn* increases gradually until it reaches a maximum value, and the value of *diam* decreases until it reaches a minimum value.

**Figure 5:**
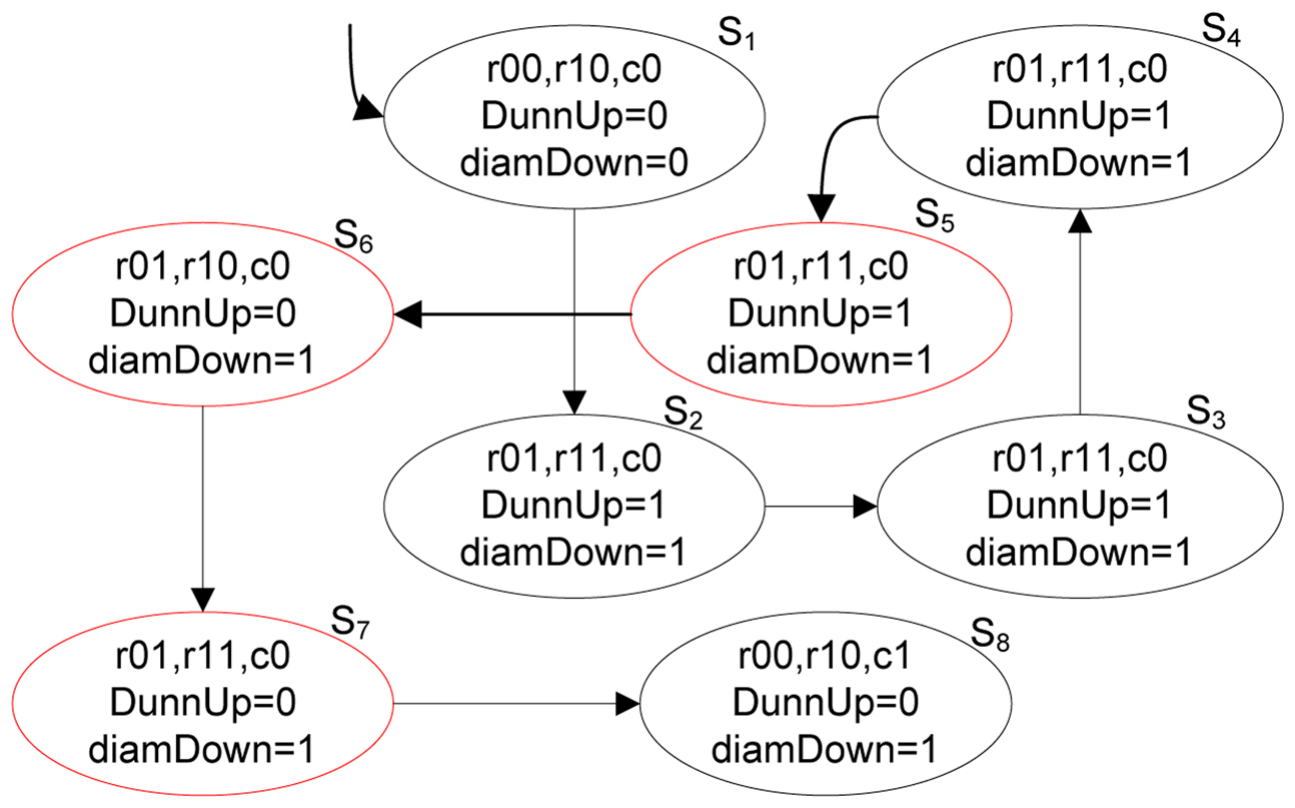
A simplified transition system of Figure S4 with atomic propositions.

We then take the same example as Figure 4 to explain these above formulas. Figure 5 presents the specific path of the clustering process in its transition system model, ignoring the “*compare*” operation because the variable values will not change after this operation. Simultaneously, Figure 5 also indicates atomic proposition *DunnUp* and *diamDown* with their truth-values in every state. In this figure, *DunnUp* starts to be false from state S_5_, so we can say that the model does not satisfy the property formula (2) based on its semantic in the transition system. In the next section, we will discuss the method of verifying properties to evaluate the validity of the clustering results and localizing the causes of the unsatisfiability automatically using model checking.

### Validity Verification Algorithms with Model Checking

It is expected that a clustering result is valid after the verification algorithm is run. If the result is not valid, we should investigate the causes of the invalid results. We divide these causes into two categories: 1) the allocation makes initial mistakes that go uncorrected until clustering terminates, and 2) the clusters of objects change with the clustering process. We will discuss methods of detecting the latter cause. This section discusses the formal method of verifying the clustering validity such that it can not only detect the validity of a clustering result but can also find and localize the objects causing the invalidity if the clustering process does not satisfy the properties to be verified.

As discussed above, for a valid clustering result, the transition system model describing the behaviors of its clustering process satisfies the properties in the CTL formula set 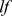 defined above. We then propose the formal verification method as follows. In a model describing a clustering process, using the idea of [33], we check whether there are traces in which formulas describing the properties to be verified are false, i.e., we check by trying to find a counterexample. We can conclude that the model satisfies these properties if there are no such traces, i.e., the corresponding clustering result is valid. If there exist traces T violating the verified properties, the clustering result is invalid. We can further localize the real causes of the violation of the properties by checking the traces C in which the verified properties are true. The causes of the violation of properties can be found by computing T\C, i.e., the causes lie along edges that belong to the error traces T but do not belong to any correct traces C.

**Figure 6:**
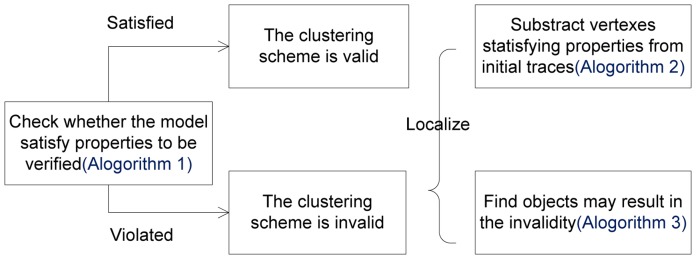
The procedure of the formal verifying method.

Figure 6 shows the procedure of our formal verifying method. If the model does not satisfy the properties to be verified, the cause of the invalidity can be obtained by subtracting the satisfied traces C from the initial detected traces T, as the invalidity may stem from only the iterations corresponding to violated vertices. The above processes are described by Algorithms 1 and 2. We can then use Algorithm 3 to obtain the objects that may influence invalid results from iterations violating properties. Therefore, the formal verifying method can analyze the validity of clustering results and can even localize the causes of invalidity.

The process of verifying clustering validity in the overall structure of our verifying method as shown in Figure 6 is described in Algorithm 1, and the algorithm for obtaining satisfied traces is presented in Algorithm 2. Method *modelCheck*(G, *p*) is used to check whether a model G satisfies the property *p* to be verified, and *modelCheck*() returns the traces satisfying the property *p*. We will perform *modelCheck*() with the model checking idea in [34], which is oriented toward CTL formulas. As mentioned above, an adequate set of temporal connectives in CTL is {AF, EU, EX}, and the corresponding CTL model checking labeling algorithm is based on connectives in this set.

Algorithm 1: validate(G, *lf*)

Input: G: state transition diagram of transition system;

*lf*: set of property formulas satisfied by system model;

Output: *real_causes*

1. *real_causes* = 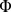;
2. **for** *i*: 1 to 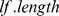 **do**
3.  
*check* : = modelcheck(G , 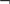 *lf*.get(*i*));
4.  
**if**(there is no trace) **then**
5.   
**continue**;
6.  
**else**
7.   
**for** *j*: 1 to 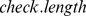 **do**
8.    T : = *check*.get(*j*);
9.    C : = T.getSatisfyTrace(T, *lf*.get(*i*), T.V);
10.    
*cause* : = T – C;
11.    
**if**( *cause* = 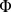) **then**
12.     
**continue**;
13.   
*real_causes* + = *cause*;
**return** *real_causes*;

Based on Algorithm 1, we can conclude that the clustering result is valid only when the model satisfies every formula in the set describing the properties, i.e., the *real_cause* that is returned from the function is null. Otherwise, traces T can be found by checking formulas describing that the verified properties are false in the model. However, not all of the vertexes in the traces violate the properties, and only the iterations corresponding to violating vertexes (states) may result in the invalidity. Algorithm 2 can localize traces C satisfying the verified properties. Then, the real reason for the violation will be detected by subtracting the traces C from the initial trace T.

Algorithm 2. getSatisfyTrace (G, *lfe*, V)

Input: G: state transition diagram of transition system;

*lfe*: property formula should be satisfied by system model;

 V: set of vertexes in state transition diagram;

Output: transition traces

1. *vtList* : = {v′ | v′
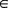
V ∧ v′. *lfe* = true};
2. *visited* : = 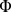;
3. *transition* : = 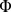;
4. **while** (vtList≠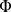) **then**
5.  
*vtList* : = *vtList* – v_j_;
6.  
**if**(v_j_ 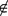 *visited*) **then**
7.   
*visited* : = *visited* ∪{v_j_};
8.   
**for** each (v_i_,v_j_)
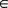
G.E **do**
9.    
*vtList* : = *vtList* ∪{v_i_};
10.    
*transition* : = *transition* ∪{(v_i_, v_j_)};
**return** *transition*;

Algorithm 2 examines the vertexes *vtList* in vertex set V satisfying the properties in the state space. While the variable *vtList* is not empty, an element v_j_ is removed. If it has not been visited before, each vertex v_i_ such that (v_i_,v_j_)
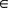
G.E is added to the *vtList*. The transition (v_i_,v_j_) is a correct transition and will be added to the set *transition*, which is returned from the function.

The complexity of Algorithm 1 largely depends on the complexity of the CTL model checking, whose complexity is linear in both the size of the transition system M and the size of property formulas Φ [35], i.e. O (|M|·|Φ|), in which |M| is depended on the transitions and states in the transition system. The transition system represents the behaviors of a clustering and the clustering process will definitely terminate, thus, the transitions in M will not be excessive. In addition, the states in our transition system correspond to a set of evaluations of variables, so the number of the states is based on the number of variables and their domains. As discussed above, the variable domains are all {0, 1}. Thus, the number of the states will not exceed 2*^t^*·2*^k^*, where *t* and *k* is number of the atomic propositions and the number of the clusters respectively. Usually *t* is very small, so 2*^t^* can be seen as a constant. When *k* is small, the exponential factor of the number will be tolerable. Therefore, the formal method of verifying the validity of clustering results based on model checking will be practical. Once the number of clusters is large enough to make the states grow exponentially, there have been some methods to reduce the states, such as symbolic model checking which can handle more than 10^20^ states, and its various refinement have pushed the state count up to more than 10^120^ [36], so its implication could not be evident.

We then take the example in Figure 5 to illustrate the operation of Algorithms 1 and 2. As mentioned above, a valid clustering process should satisfy the following properties: 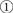 EF[*converge* ∧ *handled*→*maxDunn* ∧ *minDiam*], 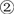
E[(*DunnUp* U *maxDunn*) ∧ (*diamDown* U *minDiam*)]. According to Algorithm 1, there are vertices whose corresponding formulas are false in the model, and we can obtain the initial trace T = (S_1_, S_2_, S_3_, S_4_, S_5_, S_6_, S_7_, S_8_). We then obtain a sub-trace C that satisfies properties based on Algorithm 2, i.e., C = (S_1_, S_2_, S_3_, S_4_). Therefore, an error trace not satisfying these properties can be obtained by subtracting trace C from the initial trace T: (S_5_, S_6_, S_7_, S_8_). Obviously, the index *Dunn* value does not increase gradually until it reaches a maximum value, and it starts to decrease from vertex S_5_. Therefore, it fails to discover the distribution pattern of this dataset effectively starting from this iteration. We conclude that this clustering process is invalid; accordingly, the corresponding clustering result is not valid.

Algorithms 1 and 2 check whether the model describing a clustering process satisfies the verified properties to analyze the validity of clustering. Furthermore, they can detect the iterations that may cause the invalidity by analyzing counterexamples. However, if we want to detect which specific objects in some iteration caused this invalidity, we should further analyze the iterations detected by the above algorithms. As mentioned in section 2, the steps in each iteration consist of comparing distances between objects and assigning objects to proper clusters. In addition, clustering is an unsupervised machine learning method, so we can analyze the correctness of the distance metric only by comparing any two adjacent assignment iterations. Furthermore, there are objects whose clusters have changed with the iterations of the clustering process, and the change is not favorable to discovering the distribution pattern of the dataset. Thus, the trend of the index value between iterations is abnormal.

Therefore, Algorithm 3 compares the iterations violating the predefined properties with their next iteration and finally returns the objects in some iteration most likely resulting in invalid clustering. First, the algorithm obtains objects whose clusters change compared with the assignment of the previous iteration as *objectSet*. If the trend of index values is normal in the next iteration (i.e., the properties to be verified in the next vertex are true), objects remaining in their clusters in the next iteration can be removed from the *objectSet* because the cluster changes of these objects are beneficial to discovering the distribution pattern of the data. Once the trend of index values is abnormal in the next iteration (i.e., the properties to be verified in the next vertex are false) and the next iteration is not the last one, objects obtained by the recursive invocation of Algorithm 3 are added to the *objectSet*; if the next iteration is the last one, new objects whose clusters will be changed when checked by the next iteration are added to the *objectSet*.

Algorithm 3. checkIteration(successor, descend)

Input: successor: set of subsequent iterations;

 descend: the trend of index value is normal or not;

Output: objectSet: set of objects leading to the invalidity;

1. *objectSet* : = getChangeObj();
2. **if** (descend =  = true) **then** // trend of index values is normal in subsequent iteration;
3.  
*nextSuccessor* : = getNextIteration();
4.  
**while** (*objectSet* is not null) **do**
5.   
**if**(object of *objectSet* is not changed in *nextSuccessor*) **then**
6.    
*objectSet* : = *objectSet* – object;
7. **else then** // trend of index values is abnormal in subsequent iteration;
8.  
*nextSuccessor* : = getNextIteration();
9.  
**if**(*nextSuccessor* <>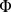) **then** // next iteration is not the last iteration
10.   
*trend* : = getNextTrend();
11.   
*nextobjSet*: = checkIteration(nextSuccessor, trend);
12.   
*objectSet* : = objectSet+nextObjSet;
13.  
**else then** // next iteration is the last iteration
14.   
**if**(object in *nextSuccessor* not in *objectSet*) **then**
15.    
*objectSet* : = *objectSet*+object;
**return** *objectSet*;

The *getChangeObj*() method obtains objects whose clusters have changed compared with the previous iteration (corresponding to the vertex), while the *getNextIteration*() method obtains the assignment of the next iteration. This algorithm compares the assignment difference of objects between iterations, so its time complexity is 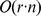, where *r* is the number of iterations checked from the current one, and *n* is the number of objects.

## Experiments

To verify the validity and applicability of the our proposed method, we will choose the following clustering algorithms, K-Means [37], DBSCAN [38], and BIRCH [39], SOM [29] which are, respectively, the most common or classic algorithms for partition clustering, density-based clustering, hierarchical clustering and neural network-based clustering. In addition, because internal indices are appropriate only for hierarchical clustering or a single clustering scheme, we will use several external indices and relative indices as the contrast methods. We choose seven common evaluation indices, Purity, Entropy, Rand, Jaccard, Dunn, DB, and RMSSTD, among which the first four are the most common external evaluation indices and the other three are commonly used relative evaluation indices. As the basis of our comparison experiments, we will conduct the experiments on eight datasets. The detailed parameters of the datasets are shown in Table 4.

**Table 4:** Experiment datasets.

No	Dataset	Number of objects	Number of attributes(Time points)
1	Iris	150	5
2	bankdata	600	11
3	Abalone	4177	8
4	Pima Indians Diabetes	768	8
5	Letter	20000	16
6	Cardiotocography	2126	23
7	Bozdech-3D7 strain data	4596	53
8	Bozdech-Hb3 strain data	4313	48

“Number of objects” indicates how many objects are in the dataset. “Number of attributes” indicates how many attributes constitute an object. The first six datasets are from machine learning datasets [40], while the rest are normalized microarray expression data from [41].

We compare our formal verification algorithm with these seven evaluation indices on the experimental datasets. First, we present the evaluation results of external indices, relative indices and our formal verification algorithm. These results are used to illustrate that our method can analyze the validity of clustering results more accurately than external indices and relative indices. We then explain that the formal verification algorithm can also check objects for invalidity if the clustering result is invalid.

**Figure 7:**
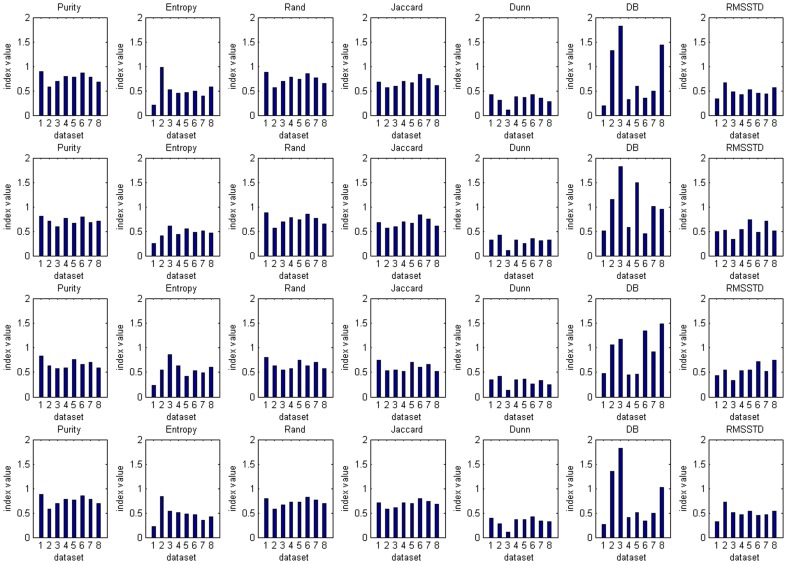
Comparison of Clustering Results for evaluation indices. K-Means results are shown in Line 1, DBSCAN results are shown in Line 2, Line 3 indicates the results of BIRCH and Line 4 indicates the results of SOM. The dataset labels are the same as Table 1.

Figure 7 presents the clustering results of eight datasets evaluated by seven common evaluation indices. For external indices, they always measure the fit between clustering and partition information. If a threshold for this fit is specified, we can determine whether a clustering result is valid by comparing its corresponding index value with the threshold, and the threshold value may affect the evaluation result. To preserve a more accurate clustering result, here we define the thresholds of Purity, Rand, Jaccard and Entropy as 0.8, 0.7, 0.7 and 0.25, respectively. Thus, Purity values larger than 0.8, Rand values larger than 0.7, Jaccard values larger than 0.7, or Entropy values smaller than 0.25 may indicate a more valid clustering result. The evaluation results of external indices are presented in Table 5. For example, the Purity and Entropy of K-Means clustering results from dataset 1 are larger than 0.8 and smaller than 0.25, respectively, and its Rand and Jaccard are both larger than 0.7, so we conclude that this result is valid. For the clustering result of K-Means on dataset 6, its Purity is larger than 0.8, its Rand and Jaccard are both larger than 0.7, so the clustering result can be seen as valid based on these indices. However, its Entropy is larger than 0.25, so the result will be regarded as invalid. With its confusion matrix of the generated clusters and pre-partition information in Table 6, we can find that there are impurities in the clusters. For example, cluster 0 is the combination of objects belonging to classes 0, 1 and 2. Therefore, we may obtain a relatively inaccurate evaluation result of K-Means on dataset 6 based only on the Purity, Rand or Jaccard index. Moreover, according to the definition of external indices, if there is no pre-partition information in the dataset, the validity of clustering results cannot be obtained based on external indices, which is the limitation of external indices.

**Table 5:** Evaluation results of external indices.

		Dataset							
Algorithms	Indices	1	2	3	4	5	6	7	8
K-Means	n	3	2	20	2	26	4	20	20
	Purity	**0.887**	0.578	0.693	**0.80**	0.774	**0.865**	0.782	0.679
	Entropy	**0.204**	0.979	0.522	0.447	0.461	0.493	0.389	0.572
	Rand	**0.874**	0.567	0.690	**0.778**	**0.739**	**0.852**	**0.771**	0.648
	Jaccard	0.683	0.559	0.588	0.692	0.658	**0.834**	**0.747**	0.609
DBSCAN	n	3	2	21	2	26	3	20	19
	Purity	**0.801**	0.707	0.593	0.759	0.663	0.786	0.683	0.712
	Entropy	**0.249**	0.402	0.608	0.442	0.544	0.479	0.512	0.469
	Rand	**0.782**	0.685	0.589	**0.732**	0.631	**0.771**	0.659	0.697
	Jaccard	**0.734**	0.629	0.544	0.678	0.607	**0.768**	0.647	0.658
BIRCH	n	3	3	10	3	12	3	18	15
	Purity	**0.836**	0.632	0.570	0.586	0.753	0.665	0.708	0.592
	Entropy	**0.228**	0.545	0.865	0.635	0.417	0.534	0.488	0.603
	Rand	**0.803**	0.628	0.544	0.579	**0.743**	0.637	**0.700**	0.576
	Jaccard	**0.751**	0.537	0.541	0.512	0.698	0.602	0.653	0.514
SOM	n	4	2	20	2	27	4	20	19
	Purity	**0.874**	0.581	0.69	0.785	0.759	**0.844**	0.78	0.693
	Entropy	**0.219**	0.83	0.542	0.506	0.482	0.461	0.355	0.424
	Rand	**0.8**	0.579	0.667	**0.719**	**0.723**	**0.815**	**0.762**	0.691
	Jaccard	**0.705**	0.572	0.608	**0.714**	0.695	**0.793**	**0.729**	0.685

n is the number of clusters generated by a clustering algorithm on a dataset. A bold value indicates a valid result evaluated by the corresponding index.

**Table 6:** Confusion matrix of K-Means clustering results on dataset 6.

	Clusters			
Pre-Partition	0	1	2	Total
**0**	1118	100	76	1294
**1**	429	70	64	563
**2**	1	3	16	20
**3**	27	122	20	169
**Total**	1655	295	144	2126

Relative indices can be used to generate a clustering validity sequence of different clustering schemes, including the same clustering algorithm on different datasets and different clustering algorithms on the same dataset, by comparing relative index values. The comparison results are shown in Table 7, which presents the clustering validity sequence of different clustering algorithms on the same dataset for every index value. We will examine a comparison result in Table 7. For example, the validity sequence K-Means>SOM>BIRCH>DBSCAN can be obtained with index *Dunn* values on dataset 1. Its corresponding index values in Figure 7 ranks: K-Means>SOM>BIRCH>DBSCAN, and the larger the index *Dunn*, the better the clustering validity. In addition, although the value *Dunn* of K-Means on dataset 6 is approaching that of K-Means on dataset 1 in Figure 7, its result is still concluded to be invalid based on the Entropy value. It is obvious that we can obtain a relative evaluation result through comparing relative index values. The result can be used to analyze which algorithm has a more reasonable and valid result. However, it is difficult to analyze the validity of a clustering result directly based only on its corresponding relative index value because they are always defined by compactness in clusters or separation between clusters, which are based on the distances between objects, and they can hardly analyze the clustering validity by setting a threshold, as with external indices. Once there is only a clustering algorithm to be verified, it is also difficult to judge its validity based only on relative index values.

**Table 7:** Evaluation results of relative indices.

	Relative Indices		
Dataset	Dunn	DB	RMSSTD
1	K-Means>SOM>BIRCH>DBSCAN	K-Means>SOM>BIRCH>DBSCAN	SOM>K-Means>BIRCH>DBSCAN
2	BIRCH>DBSCAN>K-Means>SOM	BIRCH >DBSCAN>K-Means>SOM	BIRCH>DBSCAN>K-Means>SOM
3	BIRCH>DBSCAN>K-Means>SOM	BIRCH>K-Means>DBSCAN>SOM	BIRCH>DBSCAN>K-Means>SOM
4	K-Means>SOM>BIRCH>DBSCAN	K-Means>SOM>BIRCH>DBSCAN	K-Means>>SOM>BIRCH>DBSCAN
5	BIRCH>K-Means>SOM>DBSCAN	BIRCH>SOM>K-Means>DBSCAN	K-Means>SOM>BIRCH>DBSCAN
6	K-Means>SOM>DBSCAN>BIRCH	SOM>K-Means>DBSCAN>BIRCH	K-Means>SOM>DBSCAN>BIRCH
7	K-Means>SOM>BIRCH>DBSCAN	K-Means>SOM>BIRCH>DBSCAN	K-Means>SOM>BIRCH>DBSCAN
8	DBSCAN>SOM>K-Means>BIRCH	DBSCAN>SOM>K-Means>BIRCH	DBSCAN>SOM>K-Means>BIRCH

**Figure 8:**
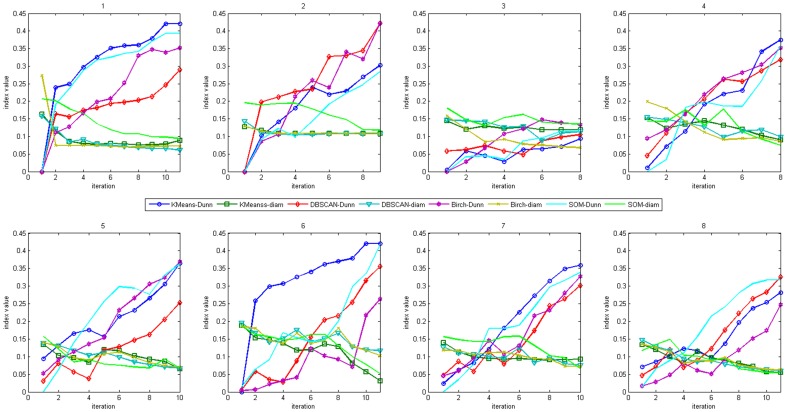
Changes of index values with iterations in executions of clustering algorithms. Every sub graph indicates one dataset. The dataset labels are also the same as Table 1.

**Figure 9:**
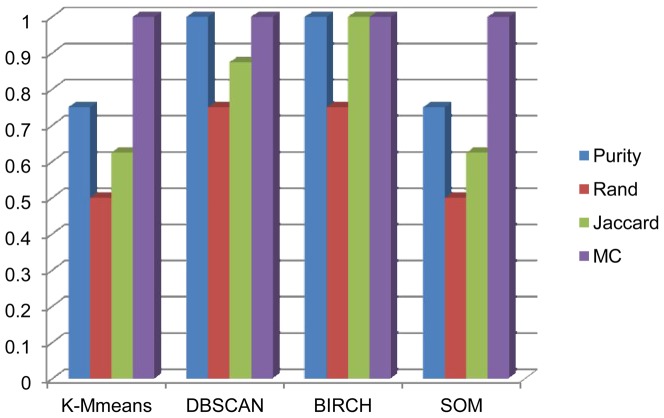
Evaluation accuracy comparison of external indices and our method.

Our method is different from the above methods based on clustering results. It focuses on a clustering process and finds properties that are satisfied by a valid clustering result. It checks whether the transition system model of a clustering process satisfies the following property formulas: 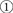 EF[*converge* ∧ *handled*→*maxDunn* ∧ *minDiam*], 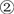 E[(*DunnUp* U *maxDunn*)∧(*diamDown* U *minDiam*)]. If there are no traces violating these properties, we can say that the clustering result to be verified is valid. First, the trends of change in index values with the iterations in execution of these four clustering algorithms are shown in Figure 8, in which the indices are those in Definition 3. Based on these trends, we can build the abstract model and measure the truth values of the property formulas of our formal verification algorithms. In Table 8, we present the verification results based on the formal verification algorithm. Using the same example as the above external experiment, the verification result of K-Means on dataset 1 is ‘valid’, while the verification result of K-Means on dataset 6 is ‘invalid’. Obviously, in contrast to relative indices, this method can directly determine the validity of a clustering result; in particular, compared with the evaluation results of external indices in Table 5, the formal algorithm can obtain a more accurate verification result, and the comparison is shown in Figure 9. The accuracy in this figure is the proportion of the evaluation results of an external index in accordance with the actual evaluations, which can be indicated by Entropy. It is obvious that our formal verification method can obtain consistent evaluation results with Entropy and more accurate evaluations than other external indices. The result reveals the accuracy of our formal verification method without pre-partition information for the dataset because it focuses on the clustering processes.

**Table 8:** Evaluation results of the formal verification algorithm.

	Clustering Algorithms			
Dataset	K-Means	DBSCAN	BIRCH	SOM
1	valid	valid	valid	valid
2	invalid	invalid	invalid	invalid
3	invalid	invalid	invalid	invalid
4	invalid	invalid	invalid	invalid
5	invalid	invalid	invalid	invalid
6	invalid	invalid	invalid	invalid
7	invalid	invalid	invalid	invalid
8	invalid	invalid	invalid	invalid

**Figure 10:**
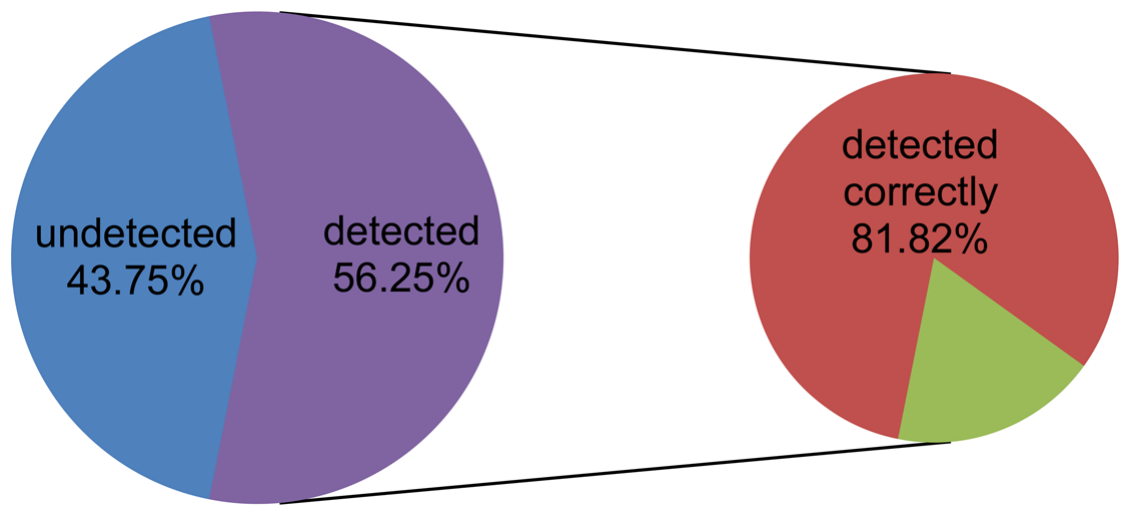
Distribution of checked objects.

**Figure 11:**
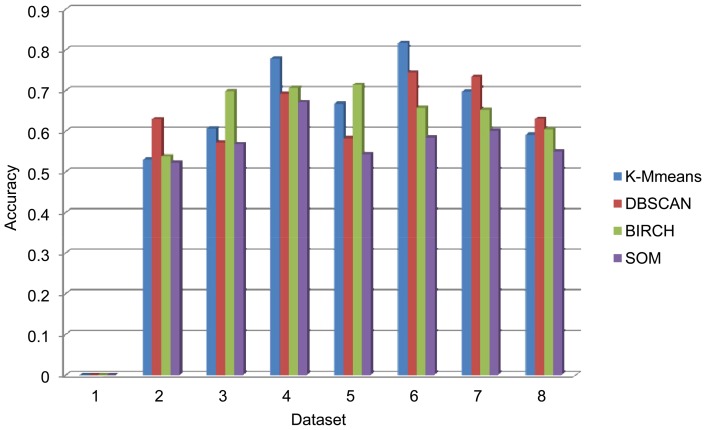
Detected accuracy of our method. The dataset labels are also the same as Table 1.

An additional advantage mentioned above is that our method can detect specific objects that may lead to the invalid result among the iterations in the traces violating the specific properties checked by Algorithm 1. To measure the accuracy of detecting objects, we will use an example. Figure 10 indicates the distribution of detected objects affecting the clustering validity of K-Means on dataset 6. As seen, the detected objects account for 56.25% of objects inconsistent with the pre-partition; i.e., we detect 56.25% of all misclassified objects. The remaining 43.75% of misclassified objects are those that are initially misallocated and that retain this error until clustering terminates, which is beyond the scope of this study. The percentage of correctly detected objects of all objects detected by Algorithm 3 that may affect clustering validity is 81.82%. We define the detection accuracy as the percentage of correct objects out of all objects detected by Algorithm 3. Figure 11 shows the verification with this definition, corresponding to the invalid results of every clustering algorithm on the experimental datasets. Because the evaluation results of dataset 1 are all valid, the verification accuracies of this dataset in Figure 11 were not computed. Most verification accuracies were larger than 60%. This finding indicates that our algorithms can detect most objects affecting the validity of clustering results.

These results show that the formal verification method we propose can not only verify the validity of clustering results but can also find and localize the causes of invalidity. Furthermore, this method is more reliable than the existing evaluation indices. However, our method still has some aspects to be improved: it should contrast different clustering results like relative indices, and it needs to detect misclassified objects caused by an initial allocation error that is retained until clustering terminates.

## Conclusion

To solve the problems in existing crisp clustering evaluation indices, we focused on the clustering processes and proposed a formal algorithm to verify the validity of clustering results based on model checking. This method can not only verify the clustering validity but, by analyzing counterexamples, can also find and localize the causes of invalidity, determine which iterations in the clustering process lead to the invalidity, and detect objects that may affect the validity of clustering algorithms. Experiments on eight datasets indicate the effectiveness and suitability of our algorithms. However, the method needs improvement: it needs to detect misclassified objects caused by an initial allocating error that is retained until the clustering terminates, and it needs to compare the effectiveness of clustering results.

## Supporting Information

Appendix S1 **CTL formulas formed by the Backus-Naur paradigm.**
(DOC)

Appendix S2 **CTL formulas with an adequate set.**
(DOC)
