# Supplementary material for: A Formal Algorithm for Verifying the Validity of Clustering Results Based on Model Checking
Source: PLoS One. 2014 Mar 7;9(3):e90109. doi: 10.1371/journal.pone.0090109 (PMC3946478; doi:10.1371/journal.pone.0090109)
Supplement: Appendix S1 — CTL formulas formed by the Backus-Naur paradigm. (DOC) [file pone.0090109.s001.doc]

ϕ ::= ⊥| ┬ | p | (¬ ϕ) | (ϕ ˄ ϕ) | (ϕ ˅ ϕ) | (ϕ → ϕ) | AX ϕ | EX ϕ | AF ϕ | EF ϕ | AG ϕ | EG ϕ | A [ ϕ U ϕ ] | E [ϕ U ϕ] |

where p is an atomic proposition formula.

The temporal connectives in CTL are a pair of symbols, such as AX, EX, AF, EF, AG, EG, etc., where A or E is a constraint on paths (A is “for all paths”, and E is “for some path”), while X (neXt) is “next state”, F (Future) is “some state in the future”, G (Globally) is “all states in the future”, and U (Until) indicates “until”.
