# Supplementary material for: A Formal Algorithm for Verifying the Validity of Clustering Results Based on Model Checking
Source: PLoS One. 2014 Mar 7;9(3):e90109. doi: 10.1371/journal.pone.0090109 (PMC3946478; doi:10.1371/journal.pone.0090109)
Supplement: Appendix S2 — CTL formulas with an adequate set. (DOC) [file pone.0090109.s002.doc]

⊥| ┬ | p | (¬ ϕ) | (ϕ ˄ ϕ) | (ϕ ˅ ϕ) | (ϕ → ϕ) | AX ϕ | EX ϕ | AF ϕ | EF ϕ | AG ϕ | EG ϕ | A [ ϕ U ϕ ] | E [ϕ U ϕ] |, and

1. AX φ = ¬ EX ¬ φ
2. A[φ1 U φ2] = ¬ (E[¬ φ2 U (¬ φ1˄¬ φ2)] ˅ EG ¬ φ2)
3. EF φ = E[┬ U φ]
4. EG φ = ¬ AF ¬ φ
5. AG φ = ¬ EF ¬ φ
